# The reality of at risk mental state services: a response to recent criticisms

**DOI:** 10.1017/S003329171900299X

**Published:** 2021-01

**Authors:** Alison R. Yung, Stephen J. Wood, Ashok Malla, Barnaby Nelson, Patrick McGorry, Jai Shah

**Affiliations:** 1 School of Health Sciences, The University of Manchester, Manchester, UK; 2 Centre for Youth Mental Health, The University of Melbourne, Melbourne, Australia; 3 Orygen, The National Centre for Excellence in Youth Mental Health, Melbourne, Australia; 4 School of Psychology, University of Birmingham, Birmingham, UK; 5 Department of Psychiatry, Douglas Research Centre, McGill University, Montreal, Canada

**Keywords:** At risk mental state, clinical high risk, prodromal, psychosis, schizophrenia, ultra high risk

## Abstract

**Background:**

In the 1990s criteria were developed to detect individuals at high and imminent risk of developing a psychotic disorder. These are known as the at risk mental state, ultra high risk or clinical high risk criteria. Individuals meeting these criteria are symptomatic and help-seeking. Services for such individuals are now found worldwide. Recently *Psychological Medicine* published two articles that criticise these services and suggest that they should be dismantled or restructured. One paper also provides recommendations on how ARMS services should be operate.

**Methods:**

In this paper we draw on the existing literature in the field and present the perspective of some ARMS clinicians and researchers.

**Results:**

Many of the critics' arguments are refuted. Most of the recommendations included in the Moritz *et al*. paper are already occurring.

**Conclusions:**

ARMS services provide management of current problems, treatment to reduce risk of onset of psychotic disorder and monitoring of mental state, including attenuated psychotic symptoms. These symptoms are associated with a range of poor outcomes. It is important to assess them and track their trajectory over time. A new approach to detection of ARMS individuals can be considered that harnesses broad youth mental health services, such as headspace in Australia, Jigsaw in Ireland and ACCESS Open Minds in Canada. Attention should also be paid to the physical health of ARMS individuals. Far from needing to be dismantled we feel that the ARMS approach has much to offer to improve the health of young people.

In the 1990s criteria were developed to detect individuals at high risk of developing a psychotic disorder, the at risk mental state (ARMS), ultra high risk (UHR) or clinical high risk criteria (Yung *et al*., 1996). The idea behind their development was that detection and intervention in these individuals may result in delay or even prevention of psychotic disorder, and reduction in distress associated with the early phases of illness. ARMS individuals are symptomatic and help-seeking, and specific services have been developed for this group. Such services are available in several continents, and management of ARMS individuals is now included in clinical guidelines across Australia (Orygen Research Centre, 2010), England (National Institute of Clinical Excellence, 2014), Canada (Addington *et al*., 2017) and Europe (Schultze-Lutter *et al*., 2015).

Recently *Psychological Medicine* published two articles that criticise services for ARMS individuals and suggest that either they be restructured and renamed (Moritz *et al*., 2019) or that they should be replaced by a ‘public health approach’ (Ajnakina *et al*., 2019). In this paper we present the perspective of some ARMS clinicians and researchers. We deal with the specific criticisms and conclude with our recommendations for the future of ARMS services.

Moritz *et al*. (2019) provide four criticisms:

(1) These services are stigmatising and cause fear of psychosis.
(2) Longer duration of untreated psychosis (DUP) is only weakly correlated with poor outcome.
(3) The criteria have poor predictive validity for psychosis.
(4) There is little evidence that interventions prevent transition to psychosis. Instead services should provide needs-based care.

In addition to argument 3 above, Ajnakina *et al*. (2019) also state that:

(5) Individuals accessing these services are not representative of all the people at risk of psychotic disorder.
(6) Sub-threshold psychotic symptoms, the hallmark of the ARMS criteria, are found in disorders such as anxiety and depression. Therefore the presence of these sub-threshold psychotic symptoms should not indicate that a person is at risk of psychotic disorder.
(7) People meeting ARMS criteria have already started the process of developing psychotic disorder and therefore intervention at this stage is too late.
(8) A public health approach to prevent psychosis would be more effective than ARMS services.

## ARMS services are stigmatising and cause fear of psychosis

Moritz *et al*. present little evidence to support this contention. They cite Corcoran *et al*. (2005), who explored the issue of the stigma that may occur by being identified as ‘at risk’. Corcoran *et al*., did not include any empirical data examining the effect of seeking help at an ARMS service or being labelled as ‘at risk’, but instead explored the issue from a hypothetical perspective. They discussed how being perceived as ‘at risk’ could vary, depending on the level of symptoms, the family's reactions and reactions of others, such as teachers. While they acknowledge that being perceived ‘at risk’ may lead to an individual feeling ‘a little bit sick’, it may also be beneficial by offering hope of treatment.

Moritz *et al*. also cite Yang *et al*. (2013) in support of their belief that ARMS services are stigmatising. However, Yang *et al*. did not directly examine this issue either. Their study was of college students who were asked about stigmatising attitudes to people labelled as having ‘psychosis risk’. If information was provided about the meaning of ‘psychosis risk’ the attitudes of participants towards individuals with this ‘diagnosis’ were no more stigmatising than their attitudes towards people with anxiety or depression.

The one study with data from ARMS individuals that Moritz *et al*. include to support their claim is Rüsch *et al*. (2015). However this study sample is mixed and includes participants deemed to be at risk of bipolar disorder and participants considered at risk of psychosis due to the presence of basic symptoms as assessed by the Schizophrenia Proneness Interview (Schultze-Lutter *et al*., 2004). Less than half met ARMS criteria, and it is not clear what was communicated to them about risk or what label was used.

It would have been useful for Moritz *et al*. to discuss the several papers that counter their assertion. For example, ARMS individuals have reported valuing the opportunity to speak about their unusual experiences (Byrne and Morrison, 2010; Welsh and Tiffin, 2012), and indeed found it important for recovery. The symptoms themselves were noted as causing a ‘fear of going mad’ (Byrne and Morrison, 2010, 2014), and disclosing them to a clinician was seen as beneficial, with reduced distress and anxiety, feeling they were going to get help, increased understanding of the symptoms and improved coping (Byrne and Morrison, 2014). Even being asked questions from a structured interview alone was found to be reassuring (Byrne and Morrison, 2014), perhaps because this helped individuals to understand that others had similar experiences. They also valued being informed about their condition (Welsh and Tiffin, 2012; Kim *et al*., 2017). Being given such information has been associated with relief, feeling validated about their experiences and encouraging hope that treatment was available (Welsh and Tiffin, 2012; Yang *et al*., 2015). In fact, stigma occurs earlier, in association with the distressing symptoms themselves (Yang *et al*., 2015), fear of being mentally ill, and self-labelling as mentally ill rather than the risk-label. Such fears and self-labelling are already present prior to seeking help and before being designated ‘at risk’ (Corcoran, 2016) and can be targeted as part of treatment for an ARMS.

In fact, the issue of potential stigma has long been recognised in ARMS work (Yung *et al*., 2010*a*), and was an argument for not reifying a diagnosis of ‘Psychosis Risk Syndrome’ in the DSM system (Yung *et al*., 2010*a*, 2010*b*). Stigma can be reduced with sensitive and careful communication that takes into account a person's context, including culture, age and cognitive capacity (Kim *et al*., 2017). Rather than telling individuals that they have a ‘diagnosis’ of ‘Attenuated Psychosis Syndrome’, the recommended approach is one of discussing current symptoms and risk, what being ‘at risk’ of psychosis means, and treatment options to reduce symptoms, distress and risk. Such discussions enable an individual to make informed decisions about treatment. It is important that the dialogue is iterative, not just a one-off communication, and that appropriate support and resources are made available for individuals and their families (Mittal *et al*., 2015). Thus, far from what Moritz *et al*. seem to think occurs across ARMS services (‘*Perhaps most importantly, the treatment is not hope-oriented. Patients are more or less told that schizophrenia is looming over them, which may stigmatize individuals who will never, in fact, develop psychosis’*), clinicians convey that ‘risk’ is not ‘disorder’, that symptoms can improve, and that recovery is possible (Corcoran, 2016). As previously noted, such communication can result in validation and relief.

To further reduce potential stigma, services for ARMS individuals should avoid being located in mental health institutions that provide services for people with established psychotic disorders. The first ARMS service, the Personal Assessment and Crisis Evaluation (PACE) Clinic, was established in a generic adolescent health service and later moved into a shopping mall (Yung *et al*., 1995). Some ARMS services are also embedded in low-stigma enhanced primary care services for young people (the *headspace* model) (McGorry *et al*., 2014). Services should have non-stigmatising names, such as ‘PACE’ (Yung *et al*., 1995), Outreach And Support in South London ‘OASIS’ (Broome *et al*., 2005), Early Detection Intervention Treatment ‘EDIT’ (Morrison *et al*., 2012), (Portland Identification and Early Referral) ‘PIER’ (McFarlane and Cook, 2006) and (Clinic for Assessment of Youth at Risk in Montréal) CAYR (Pruessner *et al*., 2017).

## Longer duration of untreated psychosis is only weakly correlated with poor outcome

Moritz *et al*. present a poorly articulated argument as to why a significant but relatively weak correlation between long DUP and poor outcome in schizophrenia patients is relevant to their criticism of ARMS services. Despite this implied linkage, they make no overt connection between the two. We speculate that they could be referring to one of the benefits of individuals engaging in ARMS services: that if a psychotic disorder occurs while being managed by such a service, then DUP should be minimal. This results in reduced time experiencing full-threshold psychotic symptoms, and hence less suffering. Such minimal DUP has been shown to be associated with improved outcomes, with a non-linear relationship. For example, a systematic review and individual patient data meta-analysis showed that individuals with a DUP less than 9 months had a substantially greater negative symptom reduction than those with a DUP of greater than 9 months (Boonstra *et al*., 2012). DUP of less than 6 months was found to be associated with better outcomes in studies from the UK (Birchwood *et al*., 2013), Poland (Cechnicki *et al*., 2014), rural China (Ran *et al*., 2018) and Canada (Dama *et al*., 2019) and less than 31 days in a study from Hong Kong (Tang *et al*., 2014). These findings suggest that very short DUP may have a marked effect on symptom reduction, highlighting this role of ARMS services for those who develop psychotic disorder.

## The criteria have poor predictive validity for psychosis

Both Moritz *et al*. and Ajnakina *et al*. make this point. We have always acknowledged that the proportion of ARMS individuals developing psychotic disorder is less than 50%. Indeed some of us were the first to report a declining transition rate (Yung *et al*., 2007) and have continued to explore potential reasons for this (Hartmann *et al*., 2016). Despite this, the ARMS criteria have fairly good validity and reliability, meaning that the ARMS can be differentiated from psychotic disorder and from mental states below the ARMS threshold, and that there tends to be agreement between clinicians about assignment of an individual into one of these groups (Woods *et al*., 2009), especially if the evaluators are trained in the use of a specialised instrument (Nelson *et al*., 2008). Further, the ARMS criteria are relatively specific for psychotic disorders (given their low incidence in the general population) (Woods *et al*., 2009; Yung and Lin, 2016). And, while a majority of ARMS individuals do not develop psychotic disorder in the short term, it is not clear how many of these ‘false positives’ might be ‘false false positives’ – that is, they would have developed psychosis but for a change in circumstance or an intervention (Yung *et al*., 1996). They may therefore develop a psychotic disorder later.

It is also important to point out that a finding of 22% of ARMS individuals developing psychotic disorder within one year (Fusar-Poli *et al*., 2012) is higher than the annual rate of development of dementia from mild cognitive impairment of 9.6% (Mitchell and Shiri-Feshki, 2009) and development of diabetes from pre-diabetes of 5–10% (Tabák *et al*., 2012). Further, people with pre-diabetes are asymptomatic, while, ARMS individuals are help-seeking, distressed and symptomatic. Indeed, ARMS individuals have been found to have higher levels of stress and lower protective factors than individuals with first episode psychosis, underlining the fact that treatment is justified (Pruessner *et al*., 2011).

Moritz *et al*. note that ARMS individuals have other poor outcomes apart from the development of psychotic disorder. Our group has previously reported this, and discussed the need to assess and target other poor outcomes, such as poor social functioning, non-psychotic disorders such as anxiety and depression, persistent attenuated psychotic symptoms and persistent negative symptoms (Yung *et al*., 2010*a*, 2010*b*; Lin *et al*., 2015; Yung *et al*., 2019). But just because other poor outcomes can occur in ARMS individuals, this does not mean that the development of psychotic disorder is any less important. In fact, the evidence that the presence of attenuated psychotic symptoms is associated with a range of poor outcomes underlines the value in assessing and treating both these symptoms and other sources of distress, regardless of whether the outcome is psychotic disorder.

Moritz *et al*. confuse their argument about predictive validity by discussing the use of self-report instruments to detect those at risk of psychotic disorder. They rightly point out that such questionnaires include terms that can be misinterpreted. They then state that, ‘We regard it as a great step forward that assessments in this area are increasingly incorporating interviews’. We encourage Moritz *et al*. to read the relevant literature in the field, which shows that structured interviews such as the Comprehensive Assessment of At Risk Mental States (Yung *et al*., 2005) and the Structured Interview for Prodromal Syndromes (Miller *et al*., 2002) have always been central to identifying ARMS individuals, and are the most widely accepted instruments for the identification of the ARMS.

## There is little evidence that interventions prevent transition to psychosis. Instead services should provide needs-based care

Moritz *et al*. draw this conclusion based on two recent network meta-analyses by Davies *et al*. (Davies *et al*. 2018*a*, 2018*b*). These studies compared interventions in the ARMS group against each other and to a ‘needs based intervention’ group. Davies *et al*. conclude that no specific pharmacological or psychological treatment was superior to needs-based intervention for reducing transition risk over 6 and 12 months (Davies *et al*., 2018*a*) or reducing attenuated psychotic symptoms (Davies *et al*., 2018*b*). These network meta-analyses have recently been criticised on methodological grounds (Nelson *et al*., 2018*a*). For example, their ‘needs-based intervention’ group combined data from multiple studies, despite the likelihood that the nature of such interventions would differ markedly across studies, due to different service systems, background treatments and the quality of the relationship between patients and the clinicians assessing need and delivering care. Additionally, most of these ‘needs-based interventions’ included active treatment components, such as supportive therapy, problem solving (Nelson *et al*., 2018*b*) and supportive monitoring (Byrne and Morrison, 2014). These are all effective in reducing distress, symptoms and risk for psychotic disorder and are provided in ARMS services. The misclassification of an active control group that included cognitive therapy in a large trial further compounded Davies *et al*.'s error (Nelson *et al*., 2018*a*). The conclusion that no treatment is effective is at odds with several meta-analyses that showed a risk reduction through use of specific treatment in ARMS patients (Stafford *et al*., 2013; van der Gaag *et al*., 2013; Hutton and Taylor, 2014; Schultze-Lutter *et al*., 2015). And while outcomes are variable in ARMS patients, symptoms and functioning tend to improve even with non-specific treatment (Nelson *et al*., 2018*b*). More research is needed into those ARMS patients who remain symptomatic and functionally impaired, perhaps with a focus on negative symptoms in this group (Yung *et al*., 2019).

Moritz *et al*. state that, rather than focusing on reducing risk for transition to psychotic disorder, ARMS services should provide needs-based care. Ajnakina *et al*. similarly fall into this falsely dichotomous position of thinking that services *either* target psychosis risk reduction *or* provide treatment based on need. In fact, ARMS services have always had such a dual purpose (Yung *et al*., 1995, 1996): amelioration, delay or prevention of psychosis onset AND management of current symptoms and psychological and functional difficulties. The problem with only focusing on needs-based treatment is that this ignores the fact the ARMS individuals may have different underlying risks for various trajectories. We need to improve our ability to differentiate these. In the meantime, we need to recognise that someone who presents with attenuated psychotic symptoms and depression likely has different underlying processes than someone with depression and no attenuated psychotic symptoms, as evidenced by the worse outcome in the former group compared to the latter (Wigman *et al*., 2012; Heinze *et al*., 2018). As noted above, the presence of attenuated psychotic symptoms is associated with a range of poor outcomes.

## Individuals accessing these services are not representative of all the people at risk of psychotic disorder

This issue raised by Ajnakina *et al*. is fundamentally a problem of the relatively poor reach of ARMS services. Ajnakina *et al*. draw this conclusion from studies from a single ARMS clinic in South London (Ajnakina *et al*., 2017). They state that individuals who attended their ARMS service and later developed psychosis were more likely to be born in the UK than those who developed psychosis without accessing their ARMS service. However, if the population of their ARMS service overall is considered, then the finding was that black and other minority ethnic background individuals were significantly *over-represented* in the ARMS group compared to the local population, with the majority being born outside the UK (Byrne *et al*., 2019). There was an even higher proportion of black service users in the first episode service compared to the background population. This study also found that black patients at the ARMS service were no more likely to make a transition to psychotic disorder than those with white British ethnicity, despite the greater than 6-fold higher incidence of psychosis among black people from the same geographical area (Fearon *et al*., 2006). Together these findings suggest that (i) their ARMS service was successful at engaging people from ethnic minorities; (ii) nonetheless, a substantial number of individuals from ethnic minority backgrounds did not access the ARMS service before developing psychosis and (iii) providing mental health care to people from ethnic minorities with an ARMS may help to reduce the risk of subsequent psychotic disorder in these vulnerable groups (Byrne *et al*., 2019).

Finally, another reason for the low number of people accessing early psychosis services via an ARMS service might be that some ARMS patients are prevented from developing psychotic disorder, as suggested by Byrne *et al*. in relation to minority ethnic group patients in the South London ARMS clinic. Rather than calling for the dismantling of their service, Ajnakina *et al*. could be advocating for more resources to increase the accessibility and acceptability of their service more widely.

## Attenuated psychotic symptoms are found in disorders such as anxiety and depression. Therefore presence of such symptoms should not indicate that a person is at risk of psychotic disorder

Individuals with anxiety and depression and attenuated psychotic symptoms (above a predefined level) will meet ARMS criteria. Such individuals may develop a psychotic disorder, persistent or recurrent mood or anxiety disorder, impaired psychosocial functioning, persistent attenuated psychotic experiences or a combination of these. They may also not develop any disorder and symptoms and functioning might resolve over time (Lin *et al*., 2015). Currently it is not possible to tell which trajectory an individual will take based on baseline clinical characteristics. We have previously argued that attenuated psychotic symptoms are not all the same, and may be indicators of three different underlying processes: (a) an expression of an underlying fundamental disturbance suggesting vulnerability to a psychotic disorder such as schizophrenia; (b) clinical ‘noise’ around an ultimately non-psychotic syndrome and (c) transient, benign or self-limiting experiences that are not associated with distress, disability or risk of disorder (Yung *et al*., 2010*b*). Research distinguishing these subtypes and examining factors that increase or decrease risk of psychotic disorder ARMS individuals is ongoing in several multi-site studies.

Ajnakina *et al*. state that, ‘The presence of psychotic symptoms in themselves *should not* be seen as an indication of the risk to making the transition’ (our emphasis). This position is contrary to decades of research that ARMS criteria do predict onset of psychotic disorder and research that shows that anxiety and depression are frequently present in the prodromal phase before a first psychotic episode (Yung and McGorry, 1996). Such sentiment may result in delayed access to appropriate care for ARMS individuals, as the significance of their attenuated psychotic symptoms may not recognised.

## People meeting ARMS criteria have already started the process of developing psychotic disorder and therefore intervention at this stage is too late

This pessimistic stance of Ajnakina *et al*. also reflected in the statement of Moritz *et al*. that, ‘we still have no treatment that can justify hope in so-called prodromal individuals’ (page 4), is at odds with numerous intervention trials and meta-analyses, and the data from the South London service in relation to ethnic minorities (see above). These statements indicate that both critic groups believe that the ARMS criteria are indicative of impending psychotic disorder. This belief is also reflected in both group's use of the term ‘prodromal’, which suggests inevitable progression to psychotic disorder (Yung *et al*., 1996). Their pessimism also contradicts their earlier points – that most individuals meeting ARMS criteria will not go on to develop a psychotic disorder – and is inconsistent with the idea that ARMS individuals should receive needs-based care to relieve current problems and distress. Even if one thought that psychotic disorder is already developing, such treatment is justified to reduce suffering, and reduce DUP.

## A public health approach to prevent psychosis would be more effective than ARMS services

Ajnakina *et al*. display binary thinking in believing that the existence of ARMS services equates to the non-existence of a public health approach and that a ‘public health approach’ applies only to the general population. However ‘public health’ is much broader than this and refers to ‘prolonging life and promoting health’ (Winslow, 1920) (p. 20). Most individuals with a first episode of psychosis pass through an ARMS phase but only a minority gain access to an ARMS service (Shah *et al*., 2017). Enabling these individuals to access care at a service that could reduce their symptoms, distress and risk of developing psychotic disorder is a public health approach. More needs to be done to improve access to ARMS services.

## The recommendations of Moritz *et al*.

In contrast to Ajnakina *et al*. who believe that the time for ARMS clinics has ‘gone’, Moritz *et al*. believe that they should be renamed, that they should provide only needs-based care, that antipsychotics should not be prescribed, that a categorical view of mental illness be avoided and a staged approach be used. They call for a ‘hope-oriented’ service, which is ironic given they believe that no treatment can justify hope. In response to their recommendations:

### Services should be renamed

We agree that calling such services ‘Prodromal Clinics’ and using the term ‘prodromal’ should be avoided, for reasons described above. However a recent study found that the terms ARMS, UHR or ‘Attenuated Psychotic Symptoms’ were not thought to be stigmatising by ARMS individuals and only a minority thought these names should be changed (Kim *et al*., 2017). Clinicians viewed these terms as more stigmatising than ARMS individuals themselves. This finding is consistent with Moritz *et al*.'s (professional) opinion that ARMS services are stigmatising despite empirical evidence to the contrary when ARMS individuals themselves are asked (see above).

### Antipsychotics should not be used

Antipsychotics are not the usual treatment for ARMS individuals. Australian, Canadian and European guidelines recommend that they only be used in exceptional circumstances, such as if symptoms are severe and progressive and have not responded to psychological therapy (Orygen Research Centre, 2010; Schultze-Lutter *et al*., 2015; Addington *et al*., 2017). English guidelines do not recommend them at all (National Institute of Clinical Excellence, 2014). Despite this, their use is variable across services. Services in the US seem to prescribe antipsychotics more readily than those in Australia, the UK, Canada and Switzerland (Yung, 2010), and we agree that this is not justified in most cases.

### A categorical view of mental illness should be avoided and a staged approach used

We appreciate Moritz *et al*. endorsing the clinical staging model in psychiatry, which we first described over a decade ago (McGorry *et al*., 2006). The model attempts to determine the position of an individual along a continuum of illness, with interventions tailored accordingly. ARMS patients are considered to be at Stage 1b – recognising that they are symptomatic and in need of intervention, but that a clear cut severe diagnosable mental disorder is not present.

### Criticism of Moritz *et al*. and Ajnakina *et al*.

Both Moritz *et al*. and Ajnakina *et al*. conflate first episode psychosis with schizophrenia. Moritz *et al*. claim that clinicians working in ARMS services put ‘a strong emphasis on the (relatively low) possibility of schizophrenia’ (p. 3) and that patients are told that ‘schizophrenia is looming over them’ (p. 1). Ajnakina *et al*. state that the ARMS criteria are ‘schizophrenia-light’. First episode psychosis is not the same as schizophrenia and not all individuals with a first episode of psychosis will develop the functional difficulties associated with this clinical syndrome, especially if early detection and comprehensive care is provided (Correll *et al*., 2018). Moritz *et al*., incorrectly cite papers about Early Intervention for Psychosis services (Birchwood and Macmillan, 1993; McGorry *et al*., 1996) when they refer to ARMS services, which they also incorrectly call ‘prodromal clinics’. Their section on stigma includes irrelevant and misleading citations. For example, they cite their own paper that found that fear of becoming psychotic was prevalent in individuals with obsessive compulsive disorder and depression (Miegel *et al*., 2019). This paper actually supports our above argument that fear of becoming psychotic is associated with the symptoms themselves rather than management in an ARMS service. They cite papers by Corcoran (Corcoran *et al*., 2005) and Yang (Yang *et al*., 2010) that do not contain any data from ARMS individuals and fail to cite the more recent papers from these authors (Corcoran, 2016) (Yang *et al*., 2015), both of which refute their position.

## Recommendations for the future of ARMS services

First, the assessment of attenuated psychotic symptoms is important. These symptoms are often distressing, can lead to maladaptive behaviours and their presence indicates risk for psychotic disorder and other poor outcomes. Cognitive behaviour therapy approaches have been developed specifically for such symptoms and have been found to be effective and non-stigmatising (Morrison *et al*., 2004, 2013). Second, while we acknowledge that being labelled as at risk of schizophrenia may be stigmatising, it is the symptoms themselves that seem to be more of a concern for individuals. Access to care at an ARMS service can play a role in reducing fear and stigma and providing hope for recovery. We have included recommendations for how stigma related to attending an ARMS service might be minimised. Third, a new approach to detection of ARMS individuals can be considered. This approach can harness broad youth mental health services, such as headspace in Australia Jigsaw in Ireland and ACCESS Open Minds in Canada (Illback and Bates, 2011; McGorry *et al*., 2014; Malla *et al*., 2019), where young people can seek help and could be screened for attenuated psychotic symptoms as part of a clinical assessment. This approach could lead to a greater proportion of ARMS individuals receiving specialised care for their attenuated psychotic symptoms and other needs. Fourth, attention should be paid to the physical health of ARMS individuals, who often display cardiometabolic risk factors (Carney *et al*., 2016). Finally, more research is needed into ARMS individuals who develop psychotic disorder despite receiving treatment at ARMS services. Understanding the pathophysiology and psychosocial factors of these individuals could lead to targeted hypothesis-driven interventions. Far from needing to be dismantled we feel that the ARMS approach has much to offer to improve the health of young people.
